# Pseudovirus Nanoparticles Displaying *Plasmodium* Circumsporozoite Proteins Elicited High Titers of Sporozoite-Binding Antibody

**DOI:** 10.3390/vaccines11111650

**Published:** 2023-10-27

**Authors:** Ming Xia, Pengwei Huang, Frank Vago, Wen Jiang, Ming Tan

**Affiliations:** 1 Division of Infectious Diseases, Cincinnati Children’s Hospital Medical Center, 3333 Burnet Avenue, Cincinnati, OH 45229, USA; ming.xia@cchmc.org (M.X.); pengwei.huang@cchmc.org (P.H.); 2 Department of Biological Sciences, Purdue University, West Lafayette, IN 47907, USA; fvago@purdue.edu (F.V.); jiang12@purdue.edu (W.J.); 3 Department of Pediatrics, University of Cincinnati College of Medicine, Cincinnati, OH 45267, USA

**Keywords:** malaria, *Plasmodium*, circumsporozoite protein, S_60_ nanoparticle, malaria vaccine

## Abstract

Background: malaria caused by *Plasmodium* parasites remains a public health threat. The circumsporozoite proteins (CSPs) of *Plasmodium* sporozoite play a key role in *Plasmodium* infection, serving as an excellent vaccine target. Methods: using a self-assembled S_60_ nanoparticle platform, we generated pseudovirus nanoparticles (PVNPs) displaying CSPs, named S-CSPs, for enhanced immunogenicity. Results: purified Hisx6-tagged or tag-free S-CSPs self-assembled into PVNPs that consist of a norovirus S_60_ inner shell and multiple surface-displayed CSPs. The majority of the PVNPs measured ~27 nm with some size variations, and their three-dimensional structure was modeled. The PVNP-displayed CSPs retained their glycan receptor-binding function. A mouse immunization study showed that PVNPs induced a high antibody response against CSP antigens and the PVNP-immunized mouse sera stained the CSPs of *Plasmodium* sporozoites at high titer. Conclusions and discussion: the PVNP-displayed CSPs retain their authentic antigenic feature and receptor-binding function. The CSP-specific antibody elicited by the S-CSP PVNPs binds original CSPs and potentially inhibits the attachment of *Plasmodium* sporozoites to their host cells, a key step for liver invasion by the sporozoites. Thus, S-CSP PVNPs may be an excellent vaccine candidate against malaria caused by *Plasmodium* parasites.

## 1. Introduction

Malaria, with typical symptoms of high fever, shaking chills, headache, and influenza-like illness, is a serious and occasionally lethal disease caused by unicellular *Plasmodium* parasites. According to the World Malaria Report 2021 by the World Health Organization (WHO) [1], there were an estimated 241 million clinical cases of malaria around the world in 2020, and approximately 627,000 deaths were attributed to malaria, highlighting malaria disease as a public health threat. Malaria occurs primarily in tropical and subtropical areas of the world. The African continent carries a vast majority of the global malaria burden. For example, 95% of malaria cases and 96% of malaria deaths occurred in this resource-limited continent in 2020 [1].

Malaria is a mosquito-borne sickness. *Plasmodium* parasites are transmitted to humans through bites by infected female *Anopheles* mosquitoes. A common prevention strategy against malaria is vector control, including indoor residual spraying of insecticide and the use of insecticide-treated mosquito nets, while chloroquine phosphate, quinine sulfate plus doxycycline, tetracycline, and clindamycin are frequently used as antimalarial medicines for therapeutic purposes. Artemisinin-based combination therapies (ACTs), which combine an artemisinin derivative with one or more other antimalarial drugs to enhance their effectiveness and reduce the risk of drug resistance, are currently the most commonly used treatments for malaria. These prevention and therapeutic approaches have helped to reduce the global malaria disease burden significantly. However, emergent resistances to insecticides among mosquitoes and to antimalarial drugs among *Plasmodium* parasites have become an apparent threat to global malaria control efforts [1].

Human infection and pathogenesis of *Plasmodium* parasites consist of two major stages, each representing an early proliferation in the liver and a later replication in the blood. *Plasmodium* infection typically starts with a mosquito bite, through which a limited number of *Plasmodium* sporozoites are transmitted from an infected mosquito into a human host, and the sporozoites ultimately reach the liver. The sporozoites replicate themselves in hepatocytes, forming merozoites, a process called schizogony [2,3]. The merozoites are then spread to infect erythrocytes for the second stage of proliferation [4,5], while some merozoites further develop into gametocytes [6,7]. The rapid and iterative attack on red blood cells by the merozoites results in the symptoms of malaria disease.

Less than 100 sporozoites are usually transferred from an infected mosquito to a human host during a mosquito bite [8], and the sporozoites are exposed to host antibodies while on their way to the liver of the host for the first stage of replication [9]. Thus, the sporozoites represent a bottleneck of *Plasmodium* infection and an excellent target for prophylactic and therapeutic approaches to prevent malaria disease. The sporozoite is covered by a multifunctional protein, named circumsporozoite protein (CSP) [10]. Importantly, CSP binds heparan sulfate proteoglycans (HSPGs) as receptors to facilitate liver invasion of the sporozoites [10,11,12,13]. Our previous data showed that CSP uses the αTSR region (region II plus) as the HSPG binding domain [14]. Prior studies also presented CSP as an immunodominant, protective antigen [15,16]. For example, messenger RNAs (mRNAs) expressing CSP from *Plasmodium falciparum* induced protective immune responses against malaria in mice [16]. In addition, injection of inactivated sporozoites produced protective immunity against malaria in rodents [17], monkeys [18], and humans [19] through the induction of CSP-specific antibodies that inhibited infection by sporozoites. Furthermore, T-cell epitopes were identified within CSP [20], supporting the idea that CSP is an ideal vaccine target.

Despite continued efforts for many years, an effective malaria vaccine remains lacking. The RTS,S/AS01 (RTS,S) vaccine, Mosquirix, developed by GlaxoSmithKline (GSK) [21,22], is the most advanced to date. This vaccine consists of a capsid protein of hepatitis B virus that is fused with the carboxy-terminal region and 18 NANP copies of the highly repetitive portion of the *P. falciparum* CSP [21,22]. In a phase III clinical trial, this vaccine showed low-level protective efficacy of 30% to 40% against severe malaria illness for the first year after vaccination, which, however, diminished afterward [21,22,23,24]. Other CSP-related antigens or epitopes were also explored, aiming to develop an effective malaria vaccine. For example, B- and T-cell epitopes of CSP were fused to a protein peptide that self-assembled into nanoparticles, which were further shown to elicit protective antibody and CD8^+^ T-cell responses [25]. In another study, three to twenty copies of NPNA repeat sequences from CSP were presented in the tobacco mosaic virus (TMV) platform, and this vaccine candidate induced a 10-fold higher antibody titer than that elicited by a nearly full-length CSP in mice [26], implying that a vaccine based on an optimized epitope focusing on the repeat region of CSP may be a good strategy to develop a malaria vaccine. In addition, approaches targeting other *Plasmodium* surface proteins have also been reported. For example, lipid nanoparticles that carried the carboxyl-terminal fragments of merozoite surface protein-1 (PfMSP-119 and PfMSP-Fu24) of *P. falciparum* elicited enhanced immunogenicity and induced protective immunity [27,28].

Our previous study demonstrated that the αTSR moiety corresponding to region II plus domain of CSP bound HSPGs [14]. A chimeric protein nanoparticle displaying 60 αTSR antigens through our S_60_ nanoparticle platform elicited a high titer of αTSR-specific antibody that stained *plasmodium* sporozoites. On the other hand, a recently developed mRNA vaccine candidate covering the full-length CSP provided complete protection against malaria in a mouse model [16]. These new advancements prompted us to generate and characterize new pseudovirus nanoparticles (PVNPs) displaying the nearly full-length CSP, referred to as S-CSP PVNPs, as a vaccine candidate. The PVNPs showed that CSPs bound HSPG glycans and elicited high CSP-specific antibodies that bound *Plasmodium* sporozoites at a high titer, implying that the CSP-specific antibodies elicited by S-CSP PVNPs could block CSP functions, and thus inhibit sporozoite attachment to host stellate cells in the liver, which is an important step of liver invasion by *Plasmodium* sporozoites. Therefore, S-CSP PVNPs may be a promising malaria vaccine candidate.

## 2. Materials and Methods

Plasmids for the expression of S-CSP fusion proteins. A gene region encoding the CSP, spanning from G27 to S384 with 358 amino acids of a *P. falciparum* strain (GenBank AC#: CAB38998.2) was synthesized using GenScript (Piscataway, NJ, USA) with codon-optimization to *Escherichia coli*. The gene was cloned into the previously constructed plasmid for production of the S_R69A_-VP8* protein [29], in which the VP8*-encoding fragment was changed to a CSP-encoding gene fragment. Two types of DNA constructs were created, each for the production of C-terminally Hisx6-tagged S-CSP and the tag-free S-CSP by adding a stop codon before the Hisx6-encoding sequence. In addition, another pET-24b-based construct was made for expression of C-terminally Hisx6-tagged CSP, but this failed to produce a soluble CSP without the S domain.

Recombinant protein expression and purification. Recombinant S-CSP fusion proteins were produced in ArcticExpress *E. coli* BL21 with an induction of 0.25 mM isopropyl-β-D-thiogalactopyranoside (IPTG) at 13 °C overnight, as reported previously [30,31]. Soluble Hisx6-tagged S-CSPs in the bacterial lysate were purified using HisPur^TM^ Cobalt Resin (Thermo Fisher Scientific, Waltham, MA, USA), while soluble tag-free S-CSPs were separated using an established two-step method that included a selective precipitation of the S-CSP from the bacterial lysate with 1.3 M ammonium sulfate [(NH_4_)_2_SO_4_], followed by anion exchange chromatography (see below) of the precipitant, as described previously [32].

Sodium dodecyl sulfate polyacrylamide gel electrophoresis (SDS-PAGE) and Western blot. The purified proteins were evaluated using SDS-PAGE. Protein concentrations were measured using SDS-PAGE with diluted bovine serum albumin (BSA, Bio-Rad, Tokyo, Japan) with known concentrations on the same gels. Western blots were performed to verify the protein identity using mouse sera after immunization with GST-αTSR fusion protein, as described previously [14].

Ion exchange was conducted to isolate S-CSP without a tag using the ӒKTA Fast Performance Liquid Chromatography (FPLC) system (GE Healthcare Life Sciences, Milwaukee, WI, USA) equipped with a HiPrep Q HP 16/10 column (GE Healthcare Life Sciences, Marlborough, MA, USA ), as described in our prior study [32]. First, the column was equilibrated by 7 column volumes (CVs) using buffer A (20 mM Tris buffer, pH 8.0). After the sample was loaded, the column was washed with 7 CVs of buffer A. Then, bound proteins were eluted using 8 CVs of buffer B (1 M NaCl in buffer A). 

Cesium chloride (CsCl) density gradient ultracentrifugation. This method was performed as described in our prior study [29]. An amount of 0.5 mL of the S-CSP was blended with 10 mL of CsCl solution. The mixture was then centrifuged at 288,000× *g* for 45 h using an Optima L-90K ultracentrifuge (Beckman Coulter). Then the gradient was fractionated into 23 portions. After 200× dilution in 1× phosphate buffer saline (PBS, pH 7.4), a sample of each fraction was coated on microtiter plates (Thermo Fisher Scientific). The S-CSP was detected using rabbit hyperimmune sera against norovirus VLPs.

Transmission electron microscopy (TEM). TEM was used to examine the morphology of the S-CSP PVNPs. Samples of the purified S-CSP were adhered to grids (Ted Pella, Inc., Redding, CA, USA) and stained with 1% ammonium molybdate. The grids were observed using an EM10 C2 microscope (Zeiss, Oberkochen, Germany), as reported in our prior study [29].

Glycan binding assay. Heparin sodium salt (Sigma-Aldrich, St. Louis, MO, USA) at 25 µg/mL concentration was coated on microtiter plates (Thermo Fisher Scientific). After blocking with 5% (*w*/*v*) nonfat milk in PBS, 100 µL of diluted S-CSP at indicated concentrations were added to the plates. The previously made GST-αTSR fusion protein that has been shown to bind heparin sulfate (HS) glycans [14] was included as a positive control, while both S_60_ and GST were used as negative controls. Bound proteins were detected using previously prepared mouse sera after immunization of the S_60_-αTSR [14] (1:2000 dilution) for the protein samples of S-CSP, GST-αTSR, and S_60,_ or the mouse hyperimmune sera against the GST-αTSR protein for GST [14] (1:1000 dilution). Then, horse-radish peroxidase (HRP)-conjugated secondary antibody (goat anti-mouse IgG, Thermo Fisher Scientific) at 1:5000 dilution was added to detect the bound antibody. Following incubation with HRP, signal intensities were measured with optical density at a wavelength of 450 nm as described previously [32].

Dynamic light scattering (DLS). DLS was used to measure the size distribution of the S-CSP PVNPs. An amount of 200 µL of purified S-CSP was added in a well of a clear-bottom 96-well microplate (Greiner Bio-One, Kremsmünster, Austria) and read with a DynaPro Plate Reader III (Wyatt Technology, Santa Barbara, CA, USA). All documented data were analyzed using software DYNAMICS v7.0 (Wyatt Technology, Santa Barbara, CA, USA).

Structural modeling of the S_60_-CSP PVNP. UCSF ChimeraX software (version 1.4) [33] was used to model the 3D structures of the S_60_-CSP PVNPs using the cryogenic electron-microscopy (cryo-EM) density map of the S_60_-VP8* nanoparticle [29] as a template. The two components of the S_60_-CSP PVNP were taken from the crystal structures of the inner shell of 60-valent VLPs of feline calicivirus (FCV) (PDB code: 4PB6) [34] and GII.4 norovirus [35], as well as the predicted structure of *P. falciparum* CSP (AF-P19597-F1) from the AlphaFold Protein Structure Database (https://alphafold.ebi.ac.uk/, accessed on 26 September 2013). UCSF ChimeraX was also used for the structural analysis of the S-CSP model and the production of the final images.

Mouse Immunizations. A total of 24 BALB/c mice at ~6 weeks of age (The Jackson Laboratory, Bar Harbor, ME, USA) were divided into three groups (n = 8) that were immunized with the following immunogens at 15 µg/mouse/dose: (1) S-CSP PVNPs (S-CSP); (2) Hisx6-tagged αTSR protein (αTSR) [14]; and (3) S_60_ nanoparticle (S_60_) [29] for comparisons. All immunogens were adjuvanted with aluminum salt (Imject Alum, Thermo Fisher Scientific) at 25 μL/dose (20 μg/mouse/dose) and delivered intramuscularly (IM) in the thigh muscle with a volume of 50 μL. Three immunizations were performed at 2-weekintervals. Serum samples were taken two weeks after the second immunization through the tail veins and two weeks after the final vaccination by cardiac puncture.

Specific IgG titer measurement. αTSR/S-CSP-specific IgG antibody titers were determined using enzyme-linked immunosorbent assays (ELISAs), as reported in our prior study [36]. In brief, GST-αTSR [14] or S-CSP at 5 µg/mL were coated on microtiter plates overnight at 4 °C. Following blocking with skim milk (5%, *w*/*v*), mouse sera at serially diluted concentrations were incubated with the coated antigens on the plates. The bound IgG was detected using HRP-conjugated goat anti-mouse IgG- (1:5000, MP Biomedicals, Santa Ana, CA, USA). The IgG titers specific to αTSR/S-CSP were described as maximum dilutions of sera with positive signals (OD_450_ ≥ 0.2).

Immunofluorescence Assays (IFAs). The mouse sera after immunization with the S-CSP PVNPs and S_60_ nanoparticle (negative control) were used to stain air-dried *P. falciparum* sporozoites, as described previously [37,38]. In brief, air-dried sporozoites of *P. falciparum* on slides that were kindly provided by Dr. Photini Sinnis at Johns Hopkins University were warmed to ambient temperature and blocked using 3% bovine serum albumin (BSA) in 1× PBS (pH 7.4). The sporozoites on the slides were incubated using mouse sera that were 2-fold serially (1000× to 32,000×) diluted. After washing, secondary antibody that was conjugated with fluorophore-FITC (Millipore Sigma) at 1:400 was added to detect the first antibodies. The slides were viewed with a fluorescence microscope at 20× to 40× magnifications.

Ethics statement. All mouse immunization studies were completed in compliance with the recommendations in the Guide for the Care and Use of Laboratory Animals (23a) of the National Institute of Health (NIH). All protocols were approved by the Institutional Animal Care and Use Committee (IACUC) of the Cincinnati Children’s Hospital Research Foundation (Animal Welfare Assurance No. A3108-01).

Statistical analysis. Statistical differences between two data groups were analyzed with GraphPad Prism 9.0 (GraphPad Software, Inc., La Jolla, CA, USA) using unpaired *t* test. Differences were categorized into highly significant when *p*-values were <0.01 (marked as “**”).

## 3. Results

**Figure 1:**
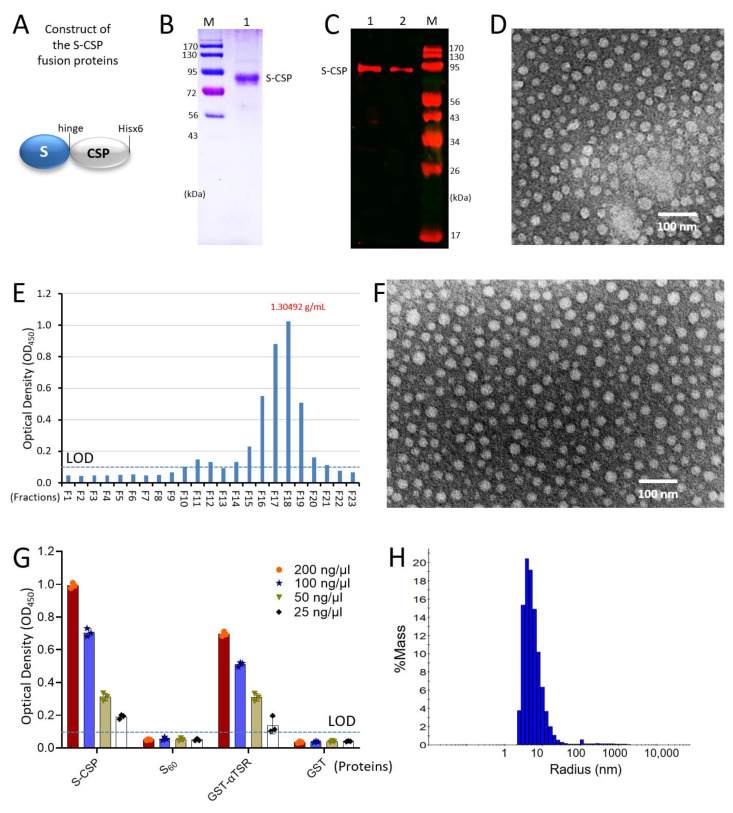
Production and characterization of the S-CSP and its PVNP formation. (**A**) Schematic construct of the S-CSP fusion protein with a Hisx6 tag at the C-terminus. S, modified norovirus S domain; hinge, the hinge region of norovirus VP1. (**B**) SDS-PAGE analysis of the resin purified S-CSP fusion protein (lane 1) showing a single major band at ~85 kDa. M, pre-stained protein standards with indicated molecular weights in kDa. (**C**) Western blot analysis of the purified S-CSP using the previously made mouse hyperimmune serum against GST-αTSR protein as detection primary antibody. Approximately 0.5 µg of S-CSP was loaded in lane 1, and ~0.25 µg was loaded in lane 2. M, protein standards with indicated molecular weights in kDa. (**D**) Negative stain TEM micrograph of the purified S-CSP showing PVNP formation. (**E**) EIA assays using the rabbit antibody against norovirus VLPs to measure the amount of S-CSP PVNPs in the 23 fractions representing different regions of the CsCl density gradient after ultracentrifugation. The Y-axis indicates the detection signal intensity in optical density (OD_450_), while the X-axis indicates the 23 fractions that were arranged from the bottom (F1) to the top (F23) of the gradient. The fraction with the highest S-CSP signal is F18. (**F**) Negative stain TEM micrograph showing S-CSP PVNPs from fraction F18. (**G**) EIA-based binding assay revealing interaction of the S-CSP with heparin sulfate using the GST-αTSR fusion protein as a positive control as well as the S_60_ nanoparticle and the GST as negative controls. The Y-axis indicates the binding signal intensity in optical density (OD_450_), while the X-axis indicates different proteins at indicated concentrations. (**H**) The particle size distribution of the resin-purified S-CSP PVNPs determined by dynamic light scattering (DLS). Dashed lines in (**E**,**G**) indicate the limits of detection (LOD).

Generation and characterization of S-CSP fusion protein with His tag. The *E. coli* expression system was used to produce soluble, C-terminally Hisx6-tagged S-CSP fusion protein (Figure 1A) at a yield of ~15 mg/liter of bacterial culture using the HisPur^TM^ Cobalt Resin. Analysis of the purified protein using SDS-PAGE revealed a major band at a molecular weight (MW) of ~85 kDa (Figure 1B) that was clearly larger than its calculated MW of ~62.9 kDa. A Western blot using the previously made antibody against GST-αTSR protein [14] was performed, showing specific signals to the purified S-CSP (Figure 1C), verifying its identity as our target protein. The observed mobility shift of the S-CSP on the SDS-PAGE implied certain unknown post-translational modifications of the S-CSP, which need to be clarified by future studies. We also attempted to generate C-terminally Hisx6-tagged CSP without the norovirus S domain through the same procedure but failed to obtain a soluble protein.

**Figure 2:**
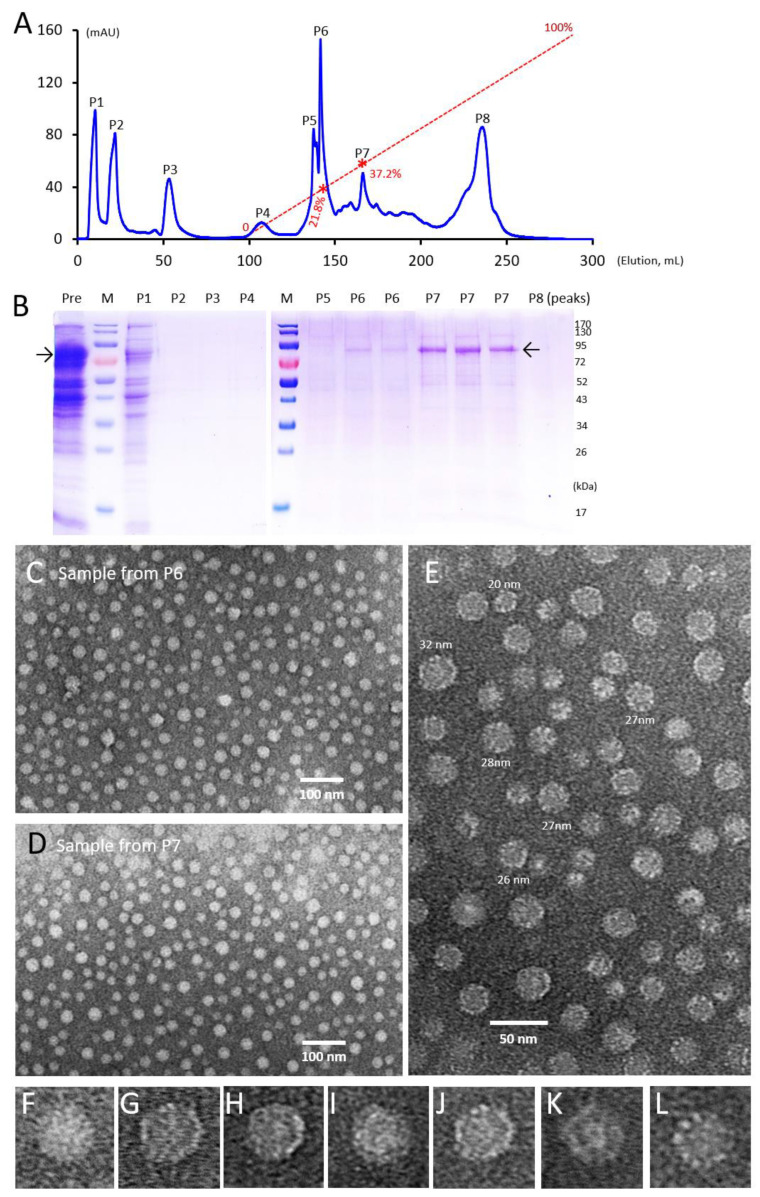
Generation and characterization of the tag-free S-CSP and its PVNP formation. (**A**) A typical elution curve from anion exchange chromatography to further purify the ammonium sulfate-precipitated S-CSP. The Y-axis indicates UV absorbances at A_280_ (mAU) of proteins in the eluents, whereas the X-axis shows the accumulated elusion volume (mL). The red dashed line shows the linear increase of buffer B (0–100%) with two red asterisks indicating the percentages of buffer B at the two elution positions of the S-CSP (21.8% for P6 and 37.3% for P7). Eight major elution peaks (P1 to P8) that were then analyzed with SDS-PAGE are denoted. (**B**) SDS-PAGE of the eight major elution peaks from the anion exchange chromatography in panel (**A**). Lane “Pre” contains the ammonium sulfate-precipitated protein that was redissolved in buffer A before loading onto the ion exchange column. Lanes “M” contains pre-stained protein standards with their molecular weights in kDa indicated on the right of the gel. The position of S-CSP fusion proteins at ~85 kDa from P6 and P7 is shown by an arrow. (**C**,**D**) Two TEM micrographs of the protein samples from P6 (**C**) and P7 (**D**) showing typical S-CSP pseudoviral nanoparticles (PVNPs). (**E**–**L**) Structural feature analyses of the S-CSP PVNPs from P7 with TEM at high magnifications. (**E**) A representative micrograph of the tag-free S-CSP PVNPs at 60,000× magnification. (**F**–**L**) Zoomed-in views of selected particles from (**E**) to enhance visualization of the structural features on the surface (**F**,**G**) and within the center (**H**–**L**) of PVNPs.

Self-formation of the S-CSP PVNPs. TEM inspection of the negatively stained Hisx6-tagged S-CSP revealed numerous nanoparticles with size variations (Figure 1D); the majority of particle sizes measured between 26 and 28 nm in diameter (Figure 2D). These data indicated that the S-CSP self-assembled into PVNPs, apparently due to the propensity of nanoparticle formation by the norovirus S domain. The particle size distribution of the Hisx6-tagged PVNPs was determined with dynamic light scattering (DLS). This revealed a collection of PVNPs at variable sizes, with major populations ranging from 5 to 30 nm (Figure 1H), consistent with our negative stain TEM data.

Density analysis of the S-CSP PVNPs. The isolated S-CSP PVNPs were then analyzed using CsCl density gradient ultracentrifugation. Following centrifugation, the gradient was separated into 23 portions, and the S-CSP fusion protein in each fraction was measured using ELISA assays with the hyperimmune serum against norovirus VLPs as a detection antibody. The data revealed that the S-CSP PVNPs accumulated in a narrow region centering at fraction 18 (Figure 1E, F18) at a density of 1.305 g/mL. Observation of fraction 18 using negative staining TEM revealed typical PVNPs (Figure 1F) similar to those observed for the resin-purified S-CSP PVNPs (Figure 1D).

Binding of the S-CSP PVNPs to heparin glycans. CSP is known to bind HSPG glycans [11,13], likely using the αTSR region as the binding domain [14]. We showed here that the S-CSP PVNPs bound heparin sulfate in a dose-dependent manner like the GST-αTSR protein (Figure 1G). These results indicated that the PVNP-displayed CSPs retain their receptor-binding function.

Generation of tag-free S-CSP PVNPs. We also developed a method to generate tag-free S-CSP PVNPs. This was achieved through a procedure consisting of two major steps. First, following protein expression in *E. coli* and lysis of the harvested bacteria by sonication, the S-CSP without a tag was precipitated by the addition of ammonium sulfate [(NH_4_)_2_SO_4_] to a final concentration of 1.3 M. The precipitated protein was resolved in 20 mM Tris buffer (pH 8.0) and was purified by ion exchange. A few elution peaks were observed (Figure 2A) and the majority of the S-CSP fusion protein was eluted in a peak (P7) equivalent to 37.2% of buffer B with a NaCl concentration of 372 mM (Figure 2A,B). A small quantity of S-CSP was eluted in peak 6 (P6), corresponding to 21.8% of buffer B with a NaCl concentration of 218 mM. Negative stain TEM inspection verified the self-formation of the tag-free S-CSP PVNPs in samples from both P6 and P7 (Figure 2C,D).

Structural features of the S-CSP PVNPs. The morphologies of the tag-free PVNPs were further studied using negative stain TEM at high magnifications between 60,000× and 80,000×. This revealed PVNPs in heterogeneous sizes that can be roughly divided into three categories: (1) about 10% of PVNPs had a diameter of ~32 nm, (2) the majority (~70%) of PVNPs had diameters between 26 and 28 nm, and (3) about 20% of the PVNPs appeared to have a diameter of ~20 nm (Figure 2E). Based on the known feature that the modified norovirus S domain has a propensity to self-assemble into a S_60_ nanoparticle with T = 1 icosahedral symmetry, and the known S_60_-VP8* nanoparticle measured ~26 nm [29], we assumed that the major population of S-CSP PVNPs with sizes between 26 and 28 nm may also be assembled with the same T = 1 icosahedral symmetry consisting of 60 S-CSP fusion proteins. Accordingly, those measuring ~32 nm may have T = 3 icosahedral symmetry, while the symmetry of those measuring ~20 nm remains unknown, but they are likely to have octahedral symmetry with 24 S-CSPs, as observed for the formation of the P_24_ nanoparticle (see Discussion).

On the other hand, micrographs at high magnifications revealed some detailed structural features of PVNPs. It was noted that some PVNPs showed multiple surface protrusions formed by the displayed CSPs (Figure 2F,G), while more PVNPs showed an inner shell with a center lumen and multiple protrusions extending outward to the surface (Figure 2H–L). The inner shells of the PVNPs exhibited different sizes with recognizable center lumens.

**Figure 3:**
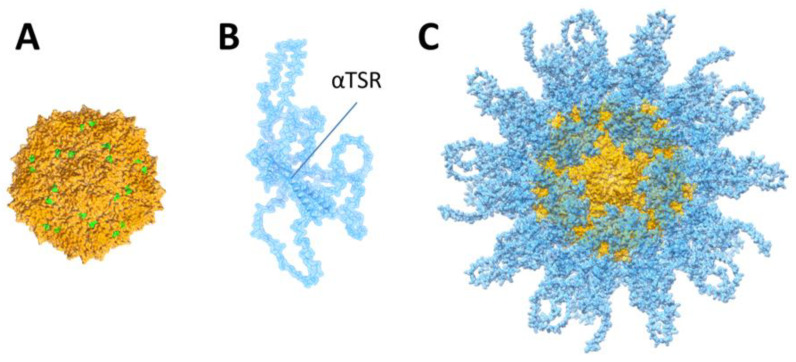
3D structural model of S_60_-CSP PVNPs. The structural model was generated using UCSF ChimeraX with the previously elucidated cryo-EM map of the S_60_-VP8* PVNP [29], in which the N-termini of 60 CSP structures were docked to the C-termini of the S domains of the S_60_-nanoparticle to replace the VP8*s. (**A**) Surface representations of the norovirus S_60_ nanoparticle, showing 60 exposed S domain termini in green. (**B**) The predicted CSP structure of *P. falciparum* in transparent surface representation from the AlphaFold Protein Structure Database (https://alphafold.ebi.ac.uk/, access on 9-27-2023, accession code of AF-P19597-F1). (**C**) Transparent surface representations of the modeled S_60_-CSP PVNP. Images in (**A**–**C**) are viewed at the 5-fold axis.

3D structural modeling of the S_60_-CSP PVNP. The major population of S-CSP PVNPs measuring ~27 nm in diameter resembled the S_60_-VP8* nanoparticle with a known structure with T = 1 icosahedral symmetry [29]. This suggested that most S-CSP PVNPs may also have T = 1 icosahedral symmetry consisting of 60 S-CSPs. While the real atomic structure of CSPs remains unknown, a predicted structure of *P. falciparum* CSP is in the AlphaFold Protein Structure Database (https://alphafold.ebi.ac.uk/; accessed on 27 September 2023, accession code: AF-P19597-F1). While this predicted structure includes the correct αTSR structure [39], the only known crystal structure of an CSP, we noted that it contains some disordered loops (Figure 3B), implying that CSP sequences are distinct from other proteins with known structures, making CSP structure difficult to predict with AlphaFold. With these uncertainties in mind, we built a 3D model of S_60_-CSP PVNP, trying to understand some structural features of the PVNP. This was achieved with the help from UCSF ChimeraX v1.4 using the previously solved cryo-EM structure of the S_60_-VP8* nanoparticle [29] as a template. The resulted model (Figure 3C) showed a similar global structure of the S_60_-CSP PVNP as seen in TEM.

**Figure 4:**
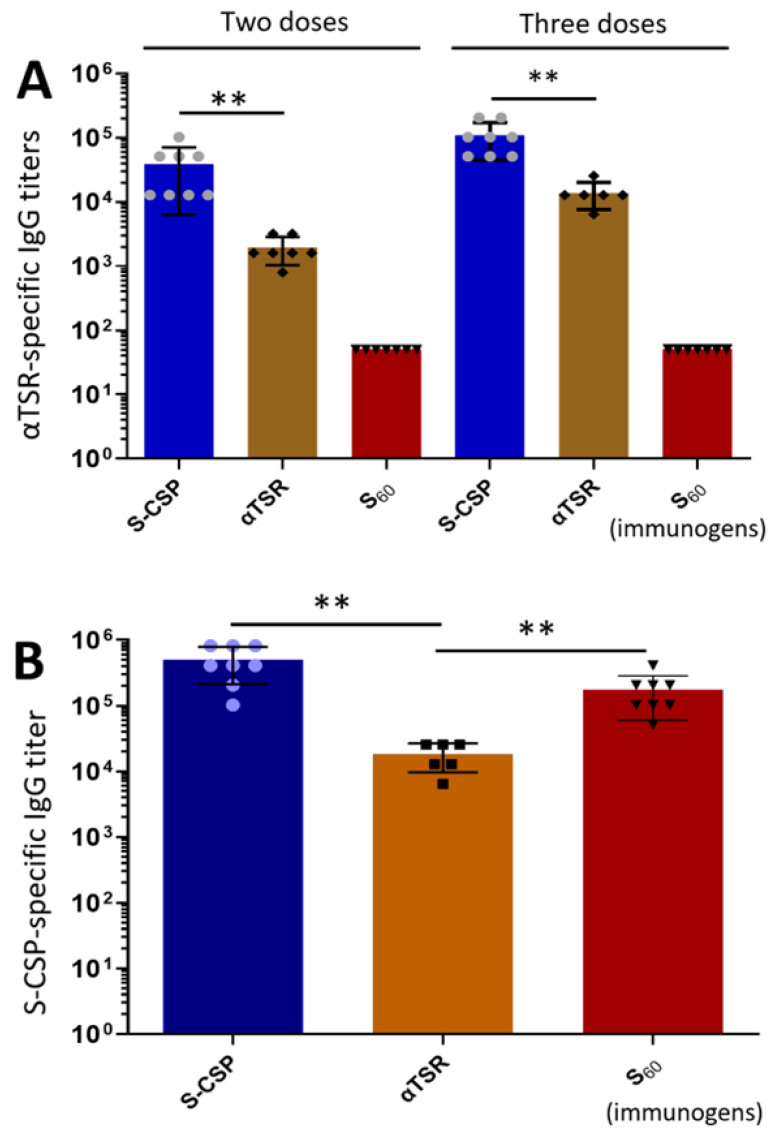
Immune responses of S-CSP PVNPs in mice. (**A**,**B**) The αTSR-specific IgG titers elicited by the S-CSP PVNPs compared with those induced by the free αTSR after two and three immunizations intramuscularly. The S_60_ nanoparticle was used as a negative control. (**B**) S-CSP-specific IgG titers elicited by the S-CSP PVNPs and the free αTSR after three immunizations. The S_60_ nanoparticle was used as a further control for comparison. In both (**A**,**B**), the Y-axes indicate αTSR-specific (**A**) or S-CSP-specific (**B**) IgG titer, while the X-axes indicate various immunogens. Statistical differences between two data groups were shown as “**” for highly significant when *p*-values were <0.01.

High antibody response of the S-CSP PVNPs toward the displayed CSP antigens. Mice were immunized with Hisx6-tagged S-CSP PVNPs and their IgG responses were measured. Because we could not generate a recombinant CSP without the S domain, we used the previously made Hisx6-tagged αTSR protein that is a part of the CSP [14] and the S60 nanoparticle [29] as a control for comparisons. Following the immunizations, the αTSR-specific IgG titers in mouse sera were determined using EIA-based assays with the previously made GST-αTSR fusion protein [14] as the capture antigen. The results showed that after two and three vaccinations, the S-CSP PVNPs induced notably higher αTSR-specific IgG titers than those induced by the αTSR protein alone (*Ps* < 0.01, Figure 4A). Specifically, following two and three injections, the αTSR-specific IgG titers elicited by the S-CSP PVNPs reached 34,800 and 108,800, 18.7 and 7.1 times higher than those induced by the αTSR protein (2057 and 15,300), respectively (*p* = 0.0096 and *p* = 0.0083). As a negative control, the S_60_ nanoparticle did not elicit detectable αTSR-specific IgG titer (<50). We also measured the IgG titers against the S-CSP PVNPs after three immunizations using the S-CSP fusion protein as the capture antigen. The results revealed that the IgG titer elicited by the S-CSP PVNPs was 499,200, 26 times higher than that induced by the free αTSR protein (19,200) (*p* = 0.0016, Figure 4B). In this case, the S_60_ nanoparticle group exhibited a high titer of 179,200, due to the fact that the immunogen and the capture antigen share a common S protein.

**Figure 5:**
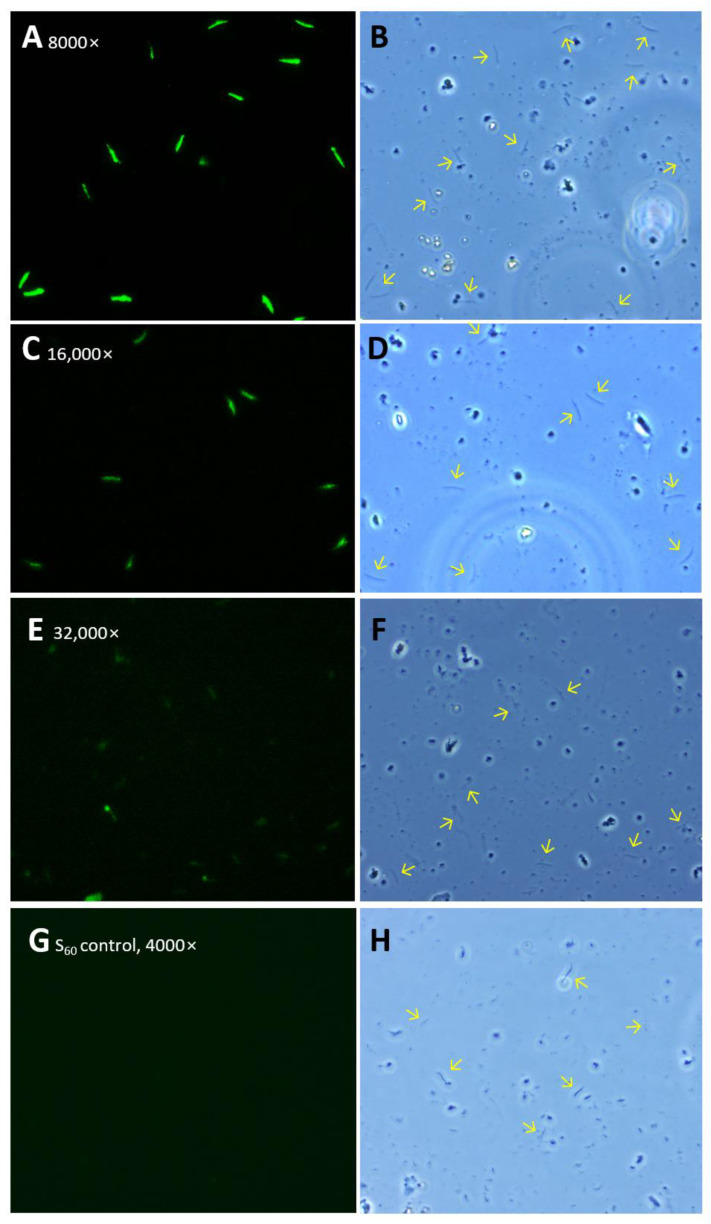
Specific staining of *P. falciparum* sporozoites by the mouse sera after immunization with S-CSP PVNPs using immunofluorescence assays (IFAs). (**A**–**F**) Representative IFA images showing the sporozoites stained by the mouse sera after three immunizations with S-CSP PVNPs. The mouse sera were diluted at 8000× (**A**,**B**), 16,000× (**C**,**D**), and 32,000× (**E**,**F**), respectively. (**G**,**H**) IFA images of sporozoites stained by 4000× diluted mouse sera after immunizations with the S_60_ nanoparticle without the CSP antigen as a negative control. Each pair of images consists of an IFA staining result (panels, **A**,**C**,**E**,**G**) and the optical view of the same field (panels, **B**,**D**,**F**,**H**). Arrows in the optical view showed some individual sporozoites that were stained in the corresponding IFA views (except the S_60_ control).

Binding of the S-CSP PVNP immunized mouse sera to *Plasmodium* sporozoites. The mouse sera after immunization with S-CSP PVNPs were further examined for their binding to the surface CSPs on *Plasmodium* sporozoites. IFAs using two mouse sera with average αTSR-specific IgG titers showed that both sera stained the air-dried *P. falciparum* sporozoites specifically without staining the “junk” around the sporozoites as shown under optical field using transmitted light (Figure 5A–F). It was noted that both sera at or below 1:8000 dilutions stained the sporozoites well (Figure 5A,B), but the staining signals became clearly weaker at or higher than 1:16,000 dilution (Figure 5C–F), suggesting that 1:16,000 dilution may be the staining limit. As a negative control, the mouse sera after immunization with the S_60_ nanoparticle without CSP antigens at 1:4000 dilution did not stain the sporozoites (Figure 5G,H). The specific staining indicated the recognition and binding of authentic CSPs on the sporozoites by the CSP-specific antibody, suggesting that the CSP-specific antibody could inhibit the function of CSPs. Therefore, S-CSP PVNPs could serve as a vaccine against malaria.

## 4. Discussion

In a previous investigation, we developed a S_60_-αTSR nanoparticle [14] using the same S_60_-nanoparticle platform technology [29] to display the αTSR that is the receptor-binding domain in the CSP of *Plasmodium* sporozoites. Based on this past success, we generated and characterized here the new S-CSP PVNPs displaying nearly the full length of the CSP antigen, aiming to develop the PVNPs into a candidate vaccine for higher efficacy. CSP has been known to play an important role in *Plasmodium* infection and has been recognized as an excellent vaccine target against malaria. The CSP antigen that we selected for the S-CSP PVNPs contained 358 amino acids covering the most epitopes of the CSP. As a result, unlike the S_60_-αTSR nanoparticle, or other known nanoparticles that display shorter fragments of CSPs [40] that induce antibodies against the short CSP regions, we anticipate that the antibody elicited by the S-CSP PVNPs will be able to bind most regions of the CSP to efficiently inhibit the function of this important surface protein and block liver invasion by the *Plasmodium* sporozoites.

The CSP with 358 residues represents the largest antigen that has been displayed by the S_60_ nanoparticle platform so far. S-CSP PVNPs can be produced by the *E. coli* system either as Hisx6-tagged PVNPs for easy purification purpose or as tag-free PVNPs to circumvent a possible regulatory issue in the future development process of S-CSP PVNPs into a useful vaccine against malaria. In both cases, similar PVNP morphologies were seen. Further investigation showed that PVNP-displayed CSPs bound their glycan receptors, and S-CSP PVNPs elicited high antibody titers in mice against the displayed CSPs. Importantly, the resulting mouse sera after immunization with S-CSP PVNPs bound the CSPs on the surface of *Plasmodium* sporozoites, supporting that S-CSP PVNPs serve as a potential vaccine candidate against infection of *Plasmodium* sporozoites.

The recombinant norovirus S domain is known for its high solubility in the *E. coli* expression system [29,32]. This specific feature may improve the solubility and folding of another proteins with low solubility when this protein is fused to the S domain. We noted in this study that the S_60_ nanoparticle platform facilitated the production of CSP as the S-CSP fusion protein, as shown by our generation of the soluble S-CSP. By contrast, we were not able to produce CSP without the S domain using the same procedure. We tried hard to generate the CSP as we intended to use it as a control in our immunization experiments and as the capture antigen for the determination of CSP-specific antibody titers. CSPs on PVNPs apparently retain their authentic folding because S-CSP PVNPs bound the glycan receptors of the CSPs. This unique feature is an advantage in applications of our S_60_ nanoparticle platform to generate various PVNPs for vaccine development against different pathogens.

Due to the lack of free CSP, we used the much shorter αTSR domain as the capture antigen in our EIA assays to determine the CSP-related IgG titers elicited by the S-CSP PVNPs. Although a high titer (108,800) after three immunizations was measured, we believed that the true CSP-specific IgG titer was higher because the αTSR domain with 67 residues represented only less than a quarter of the CSP antigen (358 residues) on the S-CSP PVNPs. Indeed, when the S-CSP fusion protein was used as the capture antigen in the EIA assays, a much higher IgG titer of 499,200 was detected. Based on these data, the true CSP-specific IgG titer should be between 108,800 and 449,200.

Unlike the uniform S_60_-αTSR nanoparticle formed by the S-αTSR fusion protein, the S-CSP PVNPs formed by the S-CSP fusion protein exhibited clear size variations, ranging from 20 to 32 nm in diameter. This scenario indicated that a small αTSR domain has less influence on the self-assembly of an S protein into a S_60_ nanoparticle [29], allowing the S-αTSR protein to assemble into a uniform S_60_-αTSR nanoparticle. However, the much larger CSPs apparently had some kind of impact on the symmetry of S nanoparticle formation, causing the observed S-CSP PVNPs to have different sizes and symmetries. Similar size and symmetric differences of norovirus VLPs have also been reported previously. For example, expression of norovirus VP1 resulted in VLPs of three different sizes, including (1) the ~30 nm VLPs with T = 1 icosahedral symmetry consisting of 60 VP1s, (2) the ~46 nm VLPs with T = 3 icosahedral symmetry containing 180 VP1s, and (3) the ~58 nm VLPs with T = 4 icosahedral symmetry containing 420 VP1s [41,42]. Based on these data, we hypothesize that ~27 nm S-CSP PVNPs may have T = 1 icosahedral symmetry containing 60 S-CSP fusion proteins, while ~32 nm PVNPs may have T = 3 icosahedral symmetry consisting of 180 S-CSP fusion proteins.

On the other hand, the symmetries of smaller S-CSP PVNPs remain to be elusive. Our previous studies on nanoparticle formation by the norovirus protruding (P) domains of VP1 revealed two other symmetries, octahedral symmetry and tetrahedral symmetry, leading to the formation of a P_24_ nanoparticle. The smaller PVNPs observed in this study could potentially be assembled through one or both these two symmetries consisting of 24 and/or 12 S-CSP fusion proteins, respectively. In addition, in our recent studies, we observed impacts on S nanoparticle formation by the displayed antigen [32,35]. In this case, S nanoparticle formation underwent certain changes, making the shell of the S nanoparticle go inward to form a compact particle core with a small center lumen [32,35]. This could also result in a smaller-sized PVNP.

We wish to point out that although the PVNPs generated in this study have different sizes and symmetries, they share the common two-component organization that consists of a core or shell formed by S domain proteins and multiple surface-displayed CSP antigens. In other words, PVNPs with size variations will not significantly affect the immunogenicity of displayed CSP antigens, because the high immune response is conferred by the polyvalent nature of PVNPs with multiple CSPs and S domains that preserve their pathogen-associated molecular patterns (PAMPs) of *Plasmodium* sporozoites and noroviruses. 

In conclusion, we have developed a scalable technology to generate S-CSP PVNPs that display the surface antigen of *Plasmodium* CSPs. S-CSP PVNPs elicited high titers of the CSP-specific antibody that binds CSPs on sporozoites of *P. falciparum.* Thus, S-CSP PVNPs, together with the S_60_-αTSR nanoparticle generated previously, should serve as good candidate vaccines against *Plasmodium* infection and malaria. Our next step will be to reach out to collaborators for further evaluation of our S-CSP PVNPs and the previously made S_60_-αTSR nanoparticle for their neutralization and protective efficacy to finally prove their usefulness.
